# Safety and efficacy of pregabalin in adolescents with fibromyalgia: a randomized, double-blind, placebo-controlled trial and a 6-month open-label extension study

**DOI:** 10.1186/s12969-016-0106-4

**Published:** 2016-07-30

**Authors:** Lesley M. Arnold, Kenneth N. Schikler, Lucinda Bateman, Tahira Khan, Lynne Pauer, Pritha Bhadra-Brown, Andrew Clair, Marci L. Chew, Joseph Scavone, on behalf of the Pregabalin Adolescent Fibromyalgia Study Group, Anthony Alario, Sushrut Sudhir Babhulkar, Ramon Berenguer, Pamela Freeman, Jeffrey Gold, Steven Goodman, Hana Jarosova, Wendy Lee, Jonathan Liss, Svetlana Lvovich, Adonis Maiquez, Nabil Morcos, Shankara Nellikunja, Murray Passo, Laura Schanberg, Vijai Prakash Sharma, Charles Spencer, Mary Toth, Sarath Chandra Mouli Veeravalli, Wen-Chin Weng

**Affiliations:** 1 University of Cincinnati College of Medicine, 260 Stetson Street, Suite 3200, Cincinnati, OH 45219 USA; 2 University of Louisville School of Medicine, Kosair Children’s Hospital, Louisville, KY USA; 3 Bateman Horne Center, Salt Lake City, UT USA; 4 Pfizer, Groton, CT USA; 5 Pfizer, New York, NY USA

**Keywords:** Juvenile fibromyalgia, Clinical trial, Pain, Pregabalin

## Abstract

**Background:**

Fibromyalgia (FM) is a common pain condition characterized by widespread musculoskeletal pain and tenderness. Pregabalin is an approved treatment for adults in the United States, but there are no approved treatments for adolescents with FM.

**Methods:**

This was a 15-week, randomized, double-blind, placebo-controlled study and 6-month open-label safety trial of flexible-dose pregabalin (75–450 mg/day) for the treatment of adolescents (12–17 years) with FM. Primary outcome was change in mean pain score at endpoint (scored from 0–10, with 24-h recall). Secondary outcomes included global assessments and measures of pain, sleep, and FM impact.

**Results:**

A total of 107 subjects were randomized to treatment (54 pregabalin, 53 placebo) and 80 completed the study (44 pregabalin, 36 placebo). Improvement in mean pain score at endpoint with pregabalin versus placebo was not statistically significant, treatment difference (95 % CI), −0.66 (−1.51, 0.18), *P* = 0.121. There were significant improvements with pregabalin versus placebo in secondary outcomes of change in pain score by week (*P* < 0.05 for 10 of 15 weeks); change in pain score at week 15 (1-week recall), treatment difference (95 % CI), −0.87 (−1.68, −0.05), *P* = 0.037; and patient global impression of change, 53.1 % versus 29.5 % very much or much improved (*P* = 0.013). Trends toward improvement with pregabalin in other secondary outcomes measuring pain, sleep, and FM impact were not significant. Safety was consistent with the known profile of pregabalin in adults with FM.

**Conclusion:**

Pregabalin did not significantly improve the mean pain score in adolescents with FM. There were significant improvements in secondary outcomes measuring pain and impression of change.

**Trial registrations:**

NCT01020474; NCT01020526.

## Background

Fibromyalgia (FM) is characterized by widespread chronic pain, sleep disturbance, and fatigue [1, 2]. FM can affect both adults and adolescents, where it is associated with significant impairment of social, emotional, and physical functioning [3, 4]. The pathophysiology of FM in both adults and adolescents is unknown; however, it is likely related to abnormalities in central nervous system pain and sensory processing [5]. At the same time there appears to be a familial component to FM, and both genetic and environmental factors likely influence its development [6, 7].

FM in adolescents is associated with significant impairment in physical functioning, lower perceived health status, and higher health care utilization compared with age-matched healthy peers [7, 8]. For most patients (>80 %), the symptoms of FM persist into young adulthood [7, 8]. Estimates of the prevalence of FM in adolescents vary markedly, from 1.0 % up to 6.2 % [9–13]. These estimates are complicated by differences in the diagnostic criteria used. Recognition and diagnosis of FM in adolescents is often challenging, which can cause frustration and anxiety in patients and their parents [7].

As FM is multi-symptomatic and consists of both neurobiological and psychosocial components, a multimodal treatment approach is recommended [9]. Cognitive behavioral therapy has been shown to be effective in improving functional disability and symptoms of depression in adolescent patients with FM [14], while exercise programs may also be an effective treatment option [15]. While there are 3 pharmacological treatments (pregabalin, duloxetine, and milnacipran) approved for adult patients with FM [16–18], there are no approved options for adolescent patients with FM. We are aware of only 1 completed pharmacological trial in adolescent patients with FM; an exploratory open-label trial of fluoxetine [19], with clinical trials of milnacipran in adolescents with FM terminated early due to low enrollment [20].

A study of pregabalin in adolescents with FM was a United States Food and Drug Administration (FDA) post-marketing requirement following approval of pregabalin for the treatment of adults with FM in the United States. Based on the evidence from clinical studies of pregabalin in adults with FM [21–23], we hypothesized that pregabalin would be safe and efficacious in reducing pain severity in adolescents with FM. To test this hypothesis, we conducted a randomized, placebo-controlled, double-blind, parallel-group study and an open-label safety study to assess the safety and efficacy of pregabalin in outpatient adolescents meeting the Yunus and Masi criteria for FM [24]. A flexible-dose range of 75–450 mg/day was utilized in the study in order to improve general tolerability by allowing a wider dose range within the flexible-dose design. The approved dose range in adults with FM in the United States is 300–450 mg/day, with a starting dose of 150 mg/day.

## Methods

### Study design

This was a 15-week, randomized, double blind, parallel-group, placebo-controlled, flexible-dose, safety, and efficacy study of pregabalin in adolescents (aged 12–17 years) with FM. The study was conducted between May 2010 and December 2014 in 36 centers, including 28 in the United States, 5 in India, 2 in Taiwan, and 1 in the Czech Republic. The study consisted of 4 phases: screen/baseline (1 week), dose optimization (3 weeks), fixed dose/maintenance (12 weeks), and follow-up/taper (1 week).

Subjects who participated in the above study were given the option to receive pregabalin in a 6-month open-label safety trial, with subjects who experienced a serious adverse event (AE) during the double-blind study excluded. The open-label study was conducted between September 2010 and June 2015 in 19 centers, including 14 in the United States, 4 in India, and 1 in the Czech Republic.

The protocols adhered to the International Ethical Guidelines for Biomedical Research Involving Human Subjects, the International Conference on Harmonisation Good Clinical Practice guidelines, and the Helsinki Declaration (2008). Subjects provided written informed consent prior to participation (ClinicalTrials.gov, NCT01020474 and NCT01020526).

### Patient population

For the double-blind study, subjects were between the ages of 12 and 17 years and met the Yunus and Masi criteria for FM [24] as follows: generalized musculoskeletal aching at ≥ 3 sites for ≥ 3 months; ≥ 5 tender points; and ≥ 3 of 10 minor criteria (chronic anxiety or tension, fatigue, nonrestorative sleep, chronic headaches, irritable bowel syndrome, subjective soft tissue swelling, numbness, pain modulation by physical activities, pain modulation by weather factors, pain modulation by anxiety or stress). If ≥ 5 of the above minor criteria were present, 4 tender points were sufficient to satisfy the criteria. At screening and randomization subjects were required to have a score of ≥ 4 on the weekly pain numeric rating scale (NRS). At randomization at least 4 pain diary entries must have been completed for the preceding 7 days.

Subjects were excluded from the double-blind study if they had pain due to other conditions that may confound assessment or self-evaluation of pain associated with FM; systemic inflammatory musculoskeletal disorders or rheumatic diseases other than FM; serious active infections; untreated endocrine disorders; prior participation in a clinical trial of pregabalin, or a history of failed treatment with pregabalin, or were taking pregabalin; unstable depressive disorders or were at risk of suicide or self-harm; serious illness or abnormality that may have increased the risk associated with study participation or interfered with interpretation of study results; an active malignancy or were immunocompromised; or a history of illicit drug or alcohol abuse within the last 2 years. Medications used for relief of pain associated with FM were to be discontinued prior to the trial; however, acetaminophen (up to 3 g/day) as rescue medication was permitted. There were no prohibited medications in the open-label study. Patients were permitted to continue to receive stable (that is, starting at least 30 days prior to randomization) non-pharmacologic therapy (such as physical or psychological therapy, massage, chiropractic care, or an exercise program) throughout the study.

### Study medication

Subjects were randomized 1:1 to receive pregabalin or matched placebo according to a computer-generated pseudorandom code using the method of random permuted blocks. Pregabalin or matched placebo was administered orally twice daily. In both studies, subjects were started at 75 mg/day from the end of week 1 and escalated at each week over a 3-week period, based on investigator assessment of safety and tolerability, to an optimized dose of 75 mg/day, 150 mg/day, 300 mg/day, or 450 mg/day. Subjects in the double-blind study received their optimized dose for the 12-week maintenance phase while subjects in the open-label study could have their dose adjusted throughout the 6 months of their study.

### Efficacy outcomes

In the double-blind study, the primary efficacy outcome was the change from baseline in mean pain score at endpoint based on the subject’s daily pain diaries (NRS), performed daily in the afternoon/evening, with a 24-h recall period. Secondary efficacy outcomes included mean pain score at each week, from daily pain diaries with a 24-h recall period; proportion of 30 % and 50 % responders (subjects with a 30 % or 50 % improvement in mean pain score from baseline at endpoint); the change in mean pain score at week 15 with a 1-week recall period; patient global impression of change (PGIC); and change from baseline in sleep quality score (11-point NRS score from the daily diary; 0 = best possible sleep to 10 = worst possible sleep) at endpoint and at each week. The study also included exploratory outcomes, which were the parent global impression of change (parent GIC), a version of the PGIC completed by the subject’s parent; and the Fibromyalgia Impact Questionnaire for children (FIQ-C), a modified version of the validated FIQ [25] that addresses the adolescent population by replacing references to work with references to daily activities [26]; higher scores indicate greater impairment.

The open-label study also assessed the change in mean pain score (NRS), with the scale completed at the clinic at baseline and weeks 3, 8, 16, and 24 (with subjects reporting their pain score for the previous week). Baseline was defined as the last score prior to treatment in the double-blind study (for subjects who had received pregabalin in the double-blind study), or as the last score prior to treatment in the open-label study (for subjects who had received placebo in the double-blind study).

### Safety evaluations

In both the double-blind and open-label studies, safety and tolerability were assessed by monitoring AEs, including clinically significant symptoms and signs, physical and neurological examinations, vital signs (blood pressure and pulse rate), body weight, edema assessments, Tanner staging, and suicidal ideation and behavior.

### Statistical analysis

For the double-blind study, an estimation approach was initially used to calculate the study sample size. As the magnitude of the pain response in the adolescent FM population was unknown, a sample size of 162 randomized subjects (81 in each treatment group) was selected so that the 95 % 2-sided confidence interval (CI) of the primary outcome would have a half-width of 0.65 with coverage probability of 80 %, assuming an estimated standard deviation (SD) of 2. During the study, the US FDA requested the implementation of a formal hypothesis-testing approach comparing the treatment groups with appropriate sample size for that objective. Under this approach, a blinded assessment of the change in mean pain score of the 95 subjects who had been randomized at that point was conducted. Based on this blinded assessment and the placebo response in adult patients with FM in prior trials of pregabalin [21–23, 27, 28], an estimated treatment difference of 1.12 was calculated. Assuming SD 2, a revised sample size of 106 was estimated to provide at least 80 % power to detect a treatment difference of 1.1 or more.

The primary analysis of the primary outcome was assessed on the full analysis set (FAS) population using analysis of covariance (ANCOVA) techniques, with terms for baseline mean pain score, center, and treatment in the model. Missing data were imputed by multiple imputation method (MI) based on distribution of baseline pain scores if subjects discontinued due to AEs, abnormal laboratory test results, or lack of efficacy; otherwise, if subjects discontinued due to other reasons, it was imputed based on distribution of post-baseline weekly mean pain scores using a Markov chain Monte Carlo method.

A number of sensitivity analyses of the primary outcome were conducted using different methods for imputation of missing data: a mixed-model repeated measures (MMRM) analysis; baseline observation carried forward (BOCF) for subjects with missing week 15 mean pain score; last observation carried forward (LOCF) for subjects with missing week 15 mean pain score; and modified baseline observation carried forward (mBOCF) for subjects with missing week 15 mean pain score, which applied the BOCF rule for subjects who discontinued due to AEs and the LOCF rule for subjects who discontinued due to any other reason.

Change in mean pain score at week 15 with a 1-week recall period, proportion of 30 % and 50 % pain responders, and FIQ-C were assessed on the FAS population using BOCF for imputation of missing data. Other secondary endpoints were assessed on the FAS population using an MMRM model, with terms of treatment, center, baseline value, visit week, and treatment-by-visit interaction. Statistical significance was assessed by Fisher’s exact test. Changes in mean pain score in the open-label study are reported using descriptive statistics.

### Post hoc efficacy analyses of patient subgroups in the double-blind study

Post hoc analyses, not specified in the study protocol, were conducted to examine the change in pain score by geographic region. Change from baseline in mean pain score at endpoint based on the subject’s daily pain diaries with a 24-h recall period and proportion of PGIC responders were assessed for the subgroups of subjects from the United States (US) and from all other countries (non-US). Analyses were on the FAS population with missing data imputed by LOCF.

## Results

### Double-blind study

#### Patient population

**Fig. 1:**
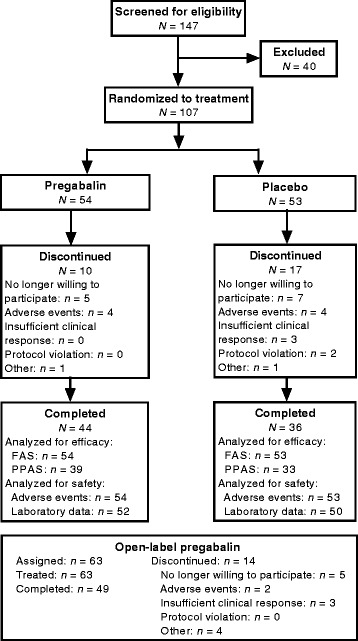
Subject disposition. *AE* adverse event, *FAS* full analysis set, *PPAS* per protocol analysis set

A total of 107 subjects were randomized to treatment (54 pregabalin, 53 placebo) and 80 completed the study (44 pregabalin, 36 placebo) (Fig. 1). The majority of subjects (86.0 %) were female and the mean age of the study population was 14.7 years (Table 1). A total of 95 (88.8 %) subjects met the 1990 American College of Rheumatology FM diagnostic criteria [2] in addition to the Yunus and Masi criteria [24].

**Table 1:** Subjects characteristics at baseline

	Double-blind trial			Open-label trial (*N* = 63)
	Pregabalin (*N* = 54)	Placebo (*N* = 53)	Total (*N* = 107)	
Hormonal status of female patients, *n*				
	48	44	92	53
Premenarchal, *n* (%)	1 (2.1)	1 (2.3)	2 (2.2)	2 (3.8)
Menarche, *n* (%)	47 (97.9)	43 (97.7)	90 (97.8)	51 (96.2)
Age, years				
Mean (SD)	14.6 (1.2)	14.7 (1.2)	14.7 (1.2)	14.8 (1.4)
Range	12–17	12–16	12–17	12–17
Age, years, *n* (%)				
12	4 (7.4)	3 (5.7)	7 (6.5)	4 (6.3)
13	5 (9.3)	6 (11.3)	11 (10.3)	8 (12.7)
14	15 (27.8)	8 (15.1)	23 (21.5)	10 (15.9)
15	18 (33.3)	21 (39.6)	39 (36.4)	19 (30.2)
16	10 (18.5)	15 (28.3)	25 (23.4)	16 (25.4)
17^a^	2 (3.7)	0	2 (1.9)	6 (9.5)
Race, *n* (%)				
White	29 (53.7)	32 (60.4)	61 (57.0)	35 (55.6)
Asian	21 (38.9)	15 (28.3)	36 (33.6)	20 (31.7)
Black	2 (3.7)	3 (5.7)	5 (4.7)	4 (6.3)
Other	2 (3.7)	3 (5.7)	5 (4.7)	4 (6.3)
Weight, kg				
Mean (SD)	60.4 (21.4)	59.7 (17.7)	60.1 (19.6)	61.6 (18.5)
Range	28.5–154.7	39.0–127.6	28.5–154.7	29.8–135.5
Height, cm				
Mean (SD)	160.1 (7.6)	162.3 (8.2)	161.2 (7.9)	161.8 (8.3)
Range	141.0–177.8	147.0–183.0	141.0–183.0	141.0–184.0
Duration of FM symptoms, years				
Mean (median)	1.7 (1.1)	2.1 (1.4)	1.9 (1.3)	0.8 (0.5)
Range	0.3–11.1	0.4–11.7	0.3–11.7	0.33.9

^a^ The study protocol was amended to include subjects aged 17 years after the study had commenced

The mean and median pregabalin doses during the maintenance phase were 244.5 mg/day and 262.3 mg/day, respectively. During this phase, 40.4 % of subjects were treated with pregabalin 450 mg/day, 15.4 % with 300 mg/day, 19.2 % with 150 mg/day, and 25.0 % with 75 mg/day. In total, 29 subjects (55.8 %) were treated with pregabalin at a dose level of 300–450 mg/day, the approved dose range for adult patients with FM in the United States.

The majority of subjects (74.1 % pregabalin, 77.4 % placebo) were receiving concomitant drug therapy during the trial. The most common concomitant treatments were ibuprofen (42.6 % pregabalin, 34.0 % placebo); paracetamol (35.2 % pregabalin, 35.8 % placebo); naproxen (18.5 % pregabalin, 13.2 % placebo); and salbutamol (11.1 % pregabalin, 13.2 % placebo).

#### Primary efficacy outcome

**Fig. 2:**
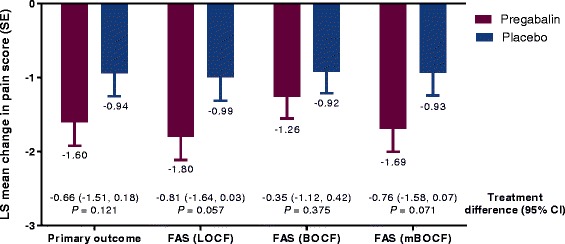
Primary efficacy outcome and sensitivity analyses in the double-blind trial *BOCF* baseline observation carried forward, *CI* confidence interval, *FAS* full analysis set; *LOCF* last observation carried forward, *mBOCF* modified baseline observation carried forward (applying BOCF rule for subjects discontinued due to adverse events and LOCF rule for subjects discontinued due to any other reason); *SE* standard error. For the primary outcome the FAS was assessed, where missing data for week 15 mean pain score were imputed based on distribution of baseline pain scores if patients discontinued due to adverse events or abnormal laboratory test results or lack of efficacy; otherwise, if subjects discontinued due to other reasons, it was imputed based on distribution of post-baseline weekly mean pain scores using Markov chain Monte Carlo method. Mean (SD) pain score at baseline was 6.94 (1.23) with pregabalin and 6.95 (1.27) with placebo

The trend toward improvement in mean pain score with pregabalin compared with placebo was not statistically significant, treatment difference (95 % CI), −0.66 (−1.51, 0.18); *P* = 0.121 (Fig. 2). The sensitivity analyses of the primary outcome showed the same trend toward improvement but were also not significant (Fig. 2).

#### Secondary efficacy outcomes

**Fig. 3:**
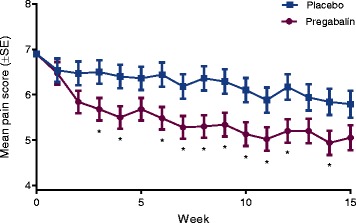
Mean pain score by week in the double-blind trial. *P < 0.05 for pregabalin compared with placebo at each week

The change in weekly mean pain score with pregabalin was significantly greater than with placebo (*P* < 0.05) for 10 of the 15 weeks assessed (Fig. 3). There was also a significantly greater change in pain score at week 15 (with a 1-week recall) with pregabalin than with placebo, treatment difference (95 % CI), −0.87 (−1.68, −0.05); *P* = 0.037.

**Fig. 4:**
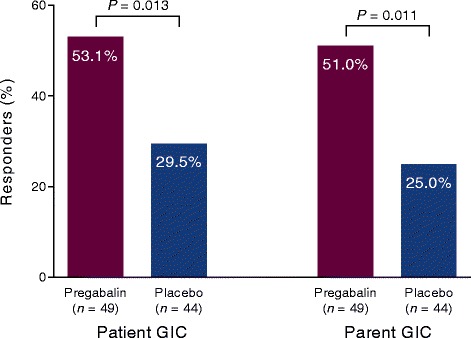
Patient global impression of change and parent global impression of change in the double-blind trial. Responders were those who were very much improved or much improved at endpoint. *P* values for pregabalin compared with placebo for each assessment. *GIC* Global impression of change

PGIC response was significantly improved with pregabalin versus placebo (*P* = 0.013), with 53.1 % of subjects much improved or very much improved at endpoint with pregabalin, compared with 29.5 % with placebo (Fig. 4). Parent GIC response was also significantly improved with pregabalin versus placebo (*P* = 0.011), with 51.0 % responders with pregabalin versus 25.0 % with placebo (Fig. 3).

No significant differences were observed in the proportions of 30 % and 50 % pain responders with pregabalin than with placebo. The proportion of 30 % responders was 33.3 % (18/54) with pregabalin and 31.4 % (16/51) with placebo, *P* = 0.830. The proportion of 50 % responders was 16.7 % (9/54) with pregabalin and 7.8 % (4/51) with placebo, *P* = 0.179.

Mean (SD) sleep quality scores at baseline were 5.8 (1.6) with pregabalin and 5.6 (2.5) with placebo. At each week and at endpoint sleep quality scores showed greater improvements for pregabalin than for placebo, but most were not statistically significant. Treatment differences (95 % CI) ranged from −1.01 (−1.73, −0.30) at week 8 to −0.17 (−0.95, 0.61) at week 15; these were significant at week 8 (*P* = 0.006) and week 10 (*P* = 0.037) but not significant at any other week or at endpoint (−0.48 [−1.02, 0.06]; *P* = 0.081). Mean (SD) FIQ-C total score at baseline was 48.44 (8.61) with pregabalin and 46.50 (10.08) with placebo. Improvements in FIQ-C total score and its subscales at endpoint with pregabalin over placebo were also not significant; the least squares mean difference (95 % CI) in FIQ-C total score compared with placebo was −2.46 (−6.87, 1.95), *P* = 0.270.

#### Post hoc efficacy analyses

There were significant differences in change in pain scores and PGIC response between subjects in the United States (*N* = 67) and subjects from other countries (*N* = 40, including 35 subjects from India, 4 from Czech Republic, and 1 from Taiwan). The mean (median) maintenance dose was 309.5 mg/day (377.5 mg/day) in US subjects and 140.5 mg/day (75.1 mg/day) in non-US subjects.

The change in mean pain score with pregabalin compared with placebo (95 % CI) was −0.78 (−1.89, 0.33), *P* = 0.1642 in US subjects, compared with −0.37 (−1.64, 0.90), *P* = 0.5600 in non-US subjects. Notably, pain improvements were greater for non-US subjects for both treatment groups; placebo-treated subjects had mean (standard error) pain reductions from baseline to week 15 of −0.52 (2.09) for US and −2.55 (1.72) for non-US, and for pregabalin-treated subjects −1.11 (2.38) in US and −2.99 (2.03) in non-US subjects. The difference in the mean change in pain score (95 % CI) between US and non-US subjects was significant with both pregabalin (1.63 [0.24, 3.02]; *P* = 0.0222) and placebo (1.83 [0.69, 2.97]; *P* = 0.0023).

There was a significant PGIC response with pregabalin in US subjects (*P* = 0.0159), with 38.7 % responders with pregabalin and 10.0 % with placebo. In non-US subjects there was no difference between pregabalin (77.8 %) and placebo (71.4 %) (*P* = 0.7035). The difference in PGIC response between US and non-US subjects was significant with both pregabalin (*P* = 0.0164) and placebo (*P* < 0.0001).

#### Safety

The most common AEs with pregabalin were dizziness and (mainly mild) nausea (Table 2). Two serious AEs (SAEs) occurred in 1 subject treated with pregabalin (cholelithiasis and major depression). Six subjects had severe AEs (3 pregabalin, 3 placebo). With pregabalin they were migraine, cholelithiasis, and major depression in 1 subject; pain; and ligament sprain. With placebo they were gastroesophageal reflux disease, vomiting, and headache in 1 subject; depressed mood and emotional disorder in 1 subject; and anemia. A total of 11 subjects (21.6 %) with pregabalin (none with placebo) experienced a weight gain of ≥7 %.

**Table 2:** Adverse events by treatment group (all causalities) in the double-blind trial

	Pregabalin (*N* = 54)	Placebo (*N* = 53)
AEs, *n*	167	132
Patients with AEs, *n* (%)	38 (70.4)	34 (64.2)
Patients with serious AEs, *n* (%)	1 (1.9)	0
Patients with severe AEs, *n* (%)	3 (5.6)	3 (5.7)
Discontinuations due to AEs, *n* (%)	4 (7.4)	4 (7.5)
Common AEs^a^, *n* (%)		
Dizziness	16 (29.6)	7 (13.2)
Nausea	12 (22.2)	5 (9.4)
Headache	10 (18.5)	10 (18.9)
Weight increased	9 (16.7)	0 (0.0)
Fatigue	8 (14.8)	4 (7.5)
Somnolence	5 (9.3)	2 (3.8)
Oropharyngeal pain	4 (7.4)	2 (3.8)
Pain in extremity	4 (7.4)	0 (0.0)
Pyrexia	4 (7.4)	3 (5.7)
Back pain	3 (5.6)	5 (9.4)
Upper respiratory tract infection	3 (5.6)	4 (7.5)
Vomiting	3 (5.6)	4 (7.5)

*AE* adverse event

^a^ Occurring in at least 5 % of subjects taking pregabalin

No clinically relevant findings were observed in physical examination, neurological examination, vital signs, laboratory test results, Tanner staging, and electrocardiogram; no positive pregnancy test results or pregnancies were reported.

#### Open-label study

A total of 63 subjects were enrolled in the open-label safety study and treated with pregabalin (40 from the United States, 20 from India, and 3 from Czech Republic), with 49 subjects completing the study. The mean (median) pregabalin dose during the maintenance phase was 254.3 (284.8) mg/day.

Improvements in pain score were observed in the open-label study both in subjects previously receiving pregabalin and in subjects previously receiving placebo. The mean (SD) pain score for all subjects was 6.7 (1.7) at baseline, 7.2 (1.2) in subjects receiving pregabalin in the double-blind study, and 6.1 (2.0) in those receiving placebo. The mean (SD) pain score at final visit was 4.6 (2.4) for all subjects together, an improvement of 2.1. In subjects who received pregabalin in the double-blind phase, the mean pain score was 4.2 (2.4) at final visit, an improvement of −2.9. In subjects who received placebo in the double-blind phase, mean pain score was 4.9 (2.4) at final visit, an improvement of −1.2.

A total of 45 subjects (71.4 %) experienced 1 or more AEs. Of these, 2 subjects (3.2 %) discontinued treatment due to AEs. A total of 3 subjects (4.8 %) experienced an SAE (migraine, appendicitis, and joint instability), and 6 subjects (9.5 %) experienced 1 or more severe AEs (appendicitis, pneumonia, arthralgia, joint instability, disturbance in attention, migraine, and mood swings). The most commonly reported AEs were dizziness (14 subjects, 22.2 %); fatigue (8 subjects, 12.7 %); headache (6 subjects, 9.5 %); and nausea, abdominal pain, and upper abdominal pain (all 5 subjects, 7.9 %). A total of 18 subjects (29.0 %) experienced a weight gain of ≥7 %. As with the double-blind study, no other clinically relevant findings were reported.

## Discussion

In this randomized, double blind, parallel-group, placebo-controlled trial in adolescents (aged 12–17 years) with FM, pregabalin did not significantly improve the primary outcome of mean change in pain score at endpoint. Certain secondary and exploratory outcomes measuring pain and impression of change were significantly improved with pregabalin. Other secondary outcomes, including FIQ-C and most sleep quality assessments, demonstrated greater improvements with pregabalin compared with placebo, but these were not statistically significant. The safety results in the double-blind and open-label studies were similar to the established safety profile in adults with FM.

While the treatment difference in the primary outcome in the double-blind study was not significant, the treatment effect on pain with pregabalin (−0.66) was within the range of those seen in prior clinical trials of pregabalin in adults with FM that had demonstrated a statistically significant treatment effect [21–23, 28]. In those trials the treatment effect (at approved doses) ranged from −0.44 [28] to −0.98 [21]. At the same time, the overall placebo response in this study was not meaningfully different from that in previous trials. The change in mean pain score at endpoint with placebo was −0.94 in this study compared with a range of −1.03 [28] to −1.40 [22] in prior trials.

In studies of pregabalin in adults with FM, patients were treated with a minimum of 300 mg/day (with the exception of the first study, which included the 150 mg/day dose level) [22], and pregabalin is approved in adults at doses of 300 or 450 mg/day [17]. In this double-blind study, almost half (44.2 %) of the subjects were treated with a maintenance dose of pregabalin that was <300 mg/day.

Post hoc analyses of the double-blind study revealed significant differences in response between subjects from the United States and those from other countries. For example, the median dose of pregabalin in non-US subjects was 75 mg/day, indicating that the majority of non-US subjects received a much lower dose than has been shown to be efficacious in adults. As these subjects had a notably smaller treatment effect with pregabalin, this might have initially suggested that utilizing a fixed, higher dose of pregabalin could have changed the outcome of this study. However, the placebo response in non-US subjects was notably large; the change in pain score at endpoint with placebo was −2.55, compared with −0.52 in US subjects. At the same time, 71.4 % of non-US subjects treated with placebo indicated that their condition was very much improved or much improved at endpoint, compared with 10.0 % of placebo-treated US subjects. This notably large placebo response suggests that a higher dose of pregabalin alone would have been unlikely to have resulted in a significant treatment difference in these subjects.

It is not clear why the placebo response was particularly high in non-US subjects. It has been suggested that the increased support and frequency of direct interaction with healthcare providers in a clinical trial can improve patients’ sense of well-being and satisfaction and contribute to a greater placebo response [29]. This may be particularly true for a condition such as FM which can benefit from a higher degree of interaction with healthcare professionals [30] and in which the lack of validation of FM as a disease has been shown to negatively affect FM patients’ sense of well-being [31]. However, it is not clear why this effect should be significantly more pronounced in the non-US subjects included in this study.

The adolescent FM population is not as well characterized as the adult FM population, and this double-blind study included a number of newer exploratory outcomes. Responses on the exploratory outcome of parent GIC were shown to be consistent with the more established method of PGIC, suggesting the parent GIC could be an effective measure in future trials in this population. While the change in FIQ-C total score with pregabalin compared with placebo in the double-blind study (−2.46) was not statistically significant, it was within the range of FIQ scores that were seen in prior studies of pregabalin in adults with FM [21, 23, 28] in which the treatment difference ranged from −2.05 [23] to −5.24 [21]. This similarity in change in FIQ-C total score between studies was despite the fact that baseline FIQ-C scores in this study (~47) were less severe than the baseline FIQ scores in prior studies (~60) [21, 23, 27]. The FIQ-C may be useful as an outcome measure in future trials in this adolescent population, although further validation is required.

The incidence and nature of AEs with pregabalin in this trial were similar to that in trials of pregabalin conducted in adults with FM [21–23, 28]. There were, however, some exceptions. The incidence of nausea in the double-blind study (22.2 %) was higher than in previous trials in adult patients in which it was ~8 %, typically less than the incidence with placebo in those trials [21, 23, 27]. The incidence of fatigue was also higher in the double-blind study than in prior trials in adults (14.8 % vs ~7.5 %). The incidence of somnolence in the double-blind study was lower than in prior trials in adults (9.3 % vs ~20 %). The reasons for these differences are not clear. FM in adolescents is commonly associated with symptoms of fatigue [7], which may make adolescent patients more prone to this AE with pregabalin. In the open-label study, there was typically a lower incidence of each common AE. The incidence of nausea was 7.9 %, similar to that seen with placebo in the double-blind trial and with pregabalin in prior trials in adults.

It was challenging to recruit patients into this trial; it required 4.5 years to enroll 107 subjects. Given the trend toward improvement in the primary efficacy outcome, it may be that the outcome could have been different if there was a larger sample size. A recent planned randomized withdrawal trial in adolescents with FM also found it challenging to recruit sufficient numbers of patients and was terminated prior to completion [20]. Clinical trials in pediatric populations are an ongoing challenge [32] and in the terminated trial, as in this one, recruitment efforts were likely limited by the underrecognition of FM in adolescents [7, 20]. Greater recognition of FM in adolescents and reassurance of patients, and their parents, that the condition can be managed, even in the absence of a definitive medical cause, may encourage families to engage with treatment recommendations [7] and to consider enrolling in clinical trials.

## Conclusions

This was the first completed large, randomized, placebo-controlled trial of a pharmacological treatment in adolescent patients with FM. No approved pharmacological treatment options are available for this patient population and, while this trial did not meet its primary efficacy outcome, improvements in secondary outcomes of pain and impression of change, together with a safety profile that was consistent with the known profile in adults with FM, suggest that patients might benefit from pharmacological treatments. We hope that this trial encourages further investigation into effective treatment options for adolescent patients with FM.

## Abbreviations

AE, adverse event; BOCF, baseline observation carried forward; CI, confidence interval; FAS, full analysis set; FDA, food and drug administration; FIQ, fibromyalgia impact questionnaire; FIQ-C, fibromyalgia impact questionnaire for children; FM, fibromyalgia; LOCF, last observation carried forward; MMRM, mixed-model repeated measure; NRS, numeric rating scale; PGIC, patient global impression of change; SAE, serious adverse event; SD, standard deviation

## Appendix

**Table 3:** Investigators^a^ in the Pregabalin Adolescent Fibromyalgia Study Group

Investigator	Institution
Anthony Alario	University of Massachusetts Memorial Medical Center, Worcester, MA, United States
Sushrut Sudhir Babhulkar	Sushrut Hospital, Research Centre & Post Graduate Institute of Orthopedics, Nagpur, India
Ramon Berenguer	Florida Medical Center & Research, Inc., Miami, FL, United States
Pamela Freeman	Rheumatology Associates of Central Florida, Orlando, FL, United States
Jeffrey Gold	Children’s Hospital Los Angeles, Los Angeles, CA, United States
Steven Goodman	Delray Research Associates, Delray Beach, FL, United States
Hana Jarosova	Bioregeneracni a rehabilitacni centrum, Ricany, Czech Republic
Wendy Lee	South Carolina Research Center, Myrtle Beach, SC, United States
Jonathan Liss	Medical Research & Health Education Foundation, Inc. Columbus, GA, United States
Svetlana Lvovich	St. Christopher's Hospital for Children, Philadelphia, PA, United States
Adonis Maiquez	Harmony Clinical Research Inc., North Miami Beach, FL, United States
Nabil Morcos	Apex Research Institute, Santa Ana, CA, United States
Shankara Nellikunja	Mallikatta Neuro Centre, Mangalore, India
Murray Passo	Medical University of South Carolina, Charleston, SC, United States
Laura Schanberg	Duke University Medical Center, Durham, NC, United States
Vijai Prakash Sharma	King George’s Medical University, Lucknow, India
Charles Spencer	Nationwide Children's Hospital, Columbus, OH, United States
Mary Toth	Akron Children's Hospital, Akron, OH, United States
Sarath Chandra Mouli Veeravalli	Krishna Institute of Medical Sciences Ltd, Secunderabad, India
Wen-Chin Weng	National Taiwan University Hospital, Taipei, Taiwan

^a^ Investigators listed in alphabetical order by last name
